# Supplementary material for: Mitochondrially tethered Mmm1 can function as a sole lipid transporter at ER–mitochondria contacts
Source: J Cell Biol. 2026 May 7;225(7):e202411196. doi: 10.1083/jcb.202411196 (PMC13151913; doi:10.1083/jcb.202411196)
Supplement: Table S1 — shows all plasmids used in this study. [file jcb_202411196_tables1.docx]

| Name | Description | Source |
| --- | --- | --- |
| pBK1 | pRS316-GPD-ChiMERA | [(Kornmann et al., 2009)](https://sciwheel.com/work/citation?ids=94472&pre=&suf=&sa=0&dbf=0) |
| pBK265 | pSOI-Vps13^GFP | [(Lang et al., 2015)](https://sciwheel.com/work/citation?ids=1255932&pre=&suf=&sa=0&dbf=0) |
| pRS314 |  | [(Sikorski and Hieter, 1989)](https://sciwheel.com/work/citation?ids=51464&pre=&suf=&sa=0&dbf=0) |
| pRS316 |  | [(Sikorski and Hieter, 1989)](https://sciwheel.com/work/citation?ids=51464&pre=&suf=&sa=0&dbf=0) |
| pRG203MX |  | [(Gnügge et al., 2016)](https://sciwheel.com/work/citation?ids=3195315&pre=&suf=&sa=0&dbf=0) |
| pTH348 | pRS316/MMM1p-Mmm1-CYC1t | This study |
| pTH349 | pRS316/MDM12p-Mdm12-CYC1t | This study |
| pTH350 | pRS316/MDM34p-Mdm34-CYC1t | This study |
| pTH351 | pRS316/MDM10p-Mdm10-CYC1t | This study |
| pTH352 | pRS314/MMM1 | This study |
| pTH353 | pRS314/MDM12 | This study |
| pTH354 | pRS314/MDM34 | This study |
| pTH355 | pRS314/MDM10 | This study |
| pTH356 | pRS314/MMM1-3FLAG-mNeonGreen | This study |
| pTH357 | pRS314/MDM12-3FLAG-mNeonGreen | This study |
| pTH358 | pRS314/MDM34-3FLAG-mNeonGreen | This study |
| pTH359 | pRS314/MMM1-3FLAG-mNeonGreen-Fis1C | This study |
| pTH360 | pRS314/Sec66N-MDM12-3FLAG-mNeonGreen-Fis1C | This study |
| pTH361 | pRS314/Ses66N-MDM34-3FLAG-mNeonGreen-Fis1C | This study |
| pTH529 | pRS316/MMM1p-Mmm1-ENO1t:MDM12p-Mdm12-SSA1t:MDM34p-Mdm34-ADH1t:MDM10p-Mdm10-PGK1t | This study |
| pTH546 | pRG203MX/TEF1p-Su9(1-69)-mTurquoise2-ENO2t:TEF2p-Kar2(1-45)-mScarletI-HDEL-TDH1t | This study |
| pTH549 | pRS314/MMM1∆(2-75)-3FLAG-mNeonGreen-Fis1C | This study |
| pTH550 | pRS314/MMM1∆(76-123)-3FLAG-mNeonGreen-Fis1C | This study |
| pTH554 | pRS314/MMM1p-Mmm1-ENO1t:MDM12p-Mdm12-SSA1t:MDM34p-Mdm34-ADH1t:MDM10p-Mdm10-PGK1t | This study |
| pTH581 | pRS314/MMM1∆(124-167)-3FLAG-mNeonGreen-Fis1C | This study |
| pTH582 | pRS314/MMM1∆(168-426)-3FLAG-mNeonGreen-Fis1C | This study |
| pTH659 | pRS314/ADH1p-Sec66N-Mmm1(168-423)-mNeonGreen-Fis1C-CYC1t | This study |
| pTH660 | pRS314/ADH1p-Sec66N-Mdm12(1-267)-mNeonGreen-Fis1C-CYC1t | This study |
| pTH661 | pRS314/ADH1p-Sec66N-Mdm34(1-190)-mNeonGreen-Fis1C-CYC1t | This study |
| pTH662 | pRS314/ADH1p-Tom70N-Mmm1(168-423)-mNeonGreen-Ubc6C-CYC1t | This study |
| pTH663 | pRS314/ADH1p-Tom70N-Mdm12(1-267)-mNeonGreen-Ubc6C-CYC1t | This study |
| pTH664 | pRS314/ADH1p-Tom70N-Mdm34(1-190)-mNeonGreen-Ubc6C-CYC1t | This study |
| pTH775 | pRS314/MMM1(F201H,V205R,I208H,I209K,F212H)-3FLAG-mNG-Fis1C | This study |
