# Supplementary material for: Mitochondrially tethered Mmm1 can function as a sole lipid transporter at ER–mitochondria contacts
Source: J Cell Biol. 2026 May 7;225(7):e202411196. doi: 10.1083/jcb.202411196 (PMC13151913; doi:10.1083/jcb.202411196)
Supplement: Table S2 — shows yeast strains used in this study. [file jcb_202411196_tables2.docx]

| Name | Genotype | Source |
| --- | --- | --- |
| ByK720 | *GPDpr::NAT-MCP1 Vps13^GFP GPDpr::KanMX6-VPS39-mCherry::HIS3* | [(John Peter et al., 2017)](https://sciwheel.com/work/citation?ids=4162214&pre=&suf=&sa=0&dbf=0) |
| ByK1665 | *GPDpr::NAT-MCP1 Vps13^GFP GPDpr::KanMX6-VPS39-mCherry::HIS3 ypt35::hph* | This study |
| ByK1472 | *GPDpr::NAT-MCP1 MDM34-mCherry::HIS3* expressing pSOI-Vps13^GFP | This study |
| ByK393 | *GPDpr::NAT-MCP1 Vps13^GFP* | [(John Peter et al., 2017)](https://sciwheel.com/work/citation?ids=4162214&pre=&suf=&sa=0&dbf=0) |
| ByK1566 | *GPDpr::NAT-MCP1 Vps13^GFP gem1::KanMX6* | This study |
| ByK1808 | *MDM12/mdm12::NAT MDM34/mdm34::KanMX6* | This study |
| ByK1809 | *MDM12/mdm12::NAT VPS13/vps13::hph* | This study |
| W303-1A | *MAT*a *ura3-1 trp1-1 leu2-3,112 his3-11,15 ade2-1 can1-100* | R. Rothstein (Columbia University, NY) |
| yTH277 | *W303-1A, mmm1∆0* | This study |
| yTH282 | *W303-1A, mdm12∆0* | This study |
| yTH287 | *W303-1A, mdm34∆0* | This study |
| yTH292 | *W303-1A, mdm10∆0* | This study |
| yTH297 | *W303-1A, mmm1∆0 mdm12∆0 mdm34∆0 mdm10∆0* | This study |
| yTH324 | *W303-1A, his3-11,15::pTH546 mmm1∆0 mdm12∆0 mdm34∆0 mdm10∆0* | This study |
