# Supplementary material for: Mitochondrially tethered Mmm1 can function as a sole lipid transporter at ER–mitochondria contacts
Source: J Cell Biol. 2026 May 7;225(7):e202411196. doi: 10.1083/jcb.202411196 (PMC13151913; doi:10.1083/jcb.202411196)
Supplement: Table S3 — shows antibodies used in this study. [file jcb_202411196_tables3.docx]

| Antibody | Species | Dilution ratio | Source (Cat. No.) |
| --- | --- | --- | --- |
| Monoclonal anti-FLAG [M2] | Mouse | 1:2000 | Merck (F1804) |
| Monoclonal anti-mNeonGreen [32F6] | Mouse | 1:2000 | Proteintech (32F6) |
| Polyclonal anti-Tom70 | Rabbit | 1:2000 | Raised in the Endo lab against *S cerevisiae* Tom70, residues 40-617 |
| IRDye 800CW-conjugated anti-mouse IgG | Goat | 1:20000 | Licor (925-32210) |
| IRDye 680RD-conjugated anti-rabbit IgG | Goat | 1:20000 | Licor (926-68071) |
