# Supplementary figures and images for: Mitochondrially tethered Mmm1 can function as a sole lipid transporter at ER–mitochondria contacts
Source: J Cell Biol. 2026 May 7;225(7):e202411196. doi: 10.1083/jcb.202411196 (PMC13151913; doi:10.1083/jcb.202411196)

**C** <sup>32</sup>P autoradiogram

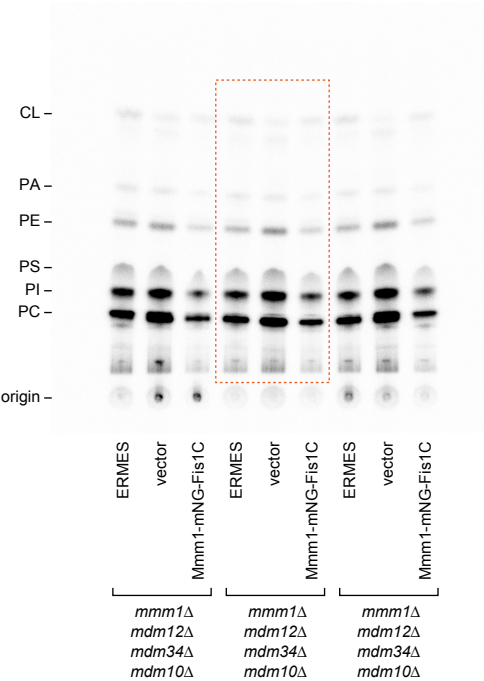

Supplement: SourceData F4 — is the source file for Fig. 4. [file jcb_202411196_sourcedataf4.pdf]

Source Data Fig. 5

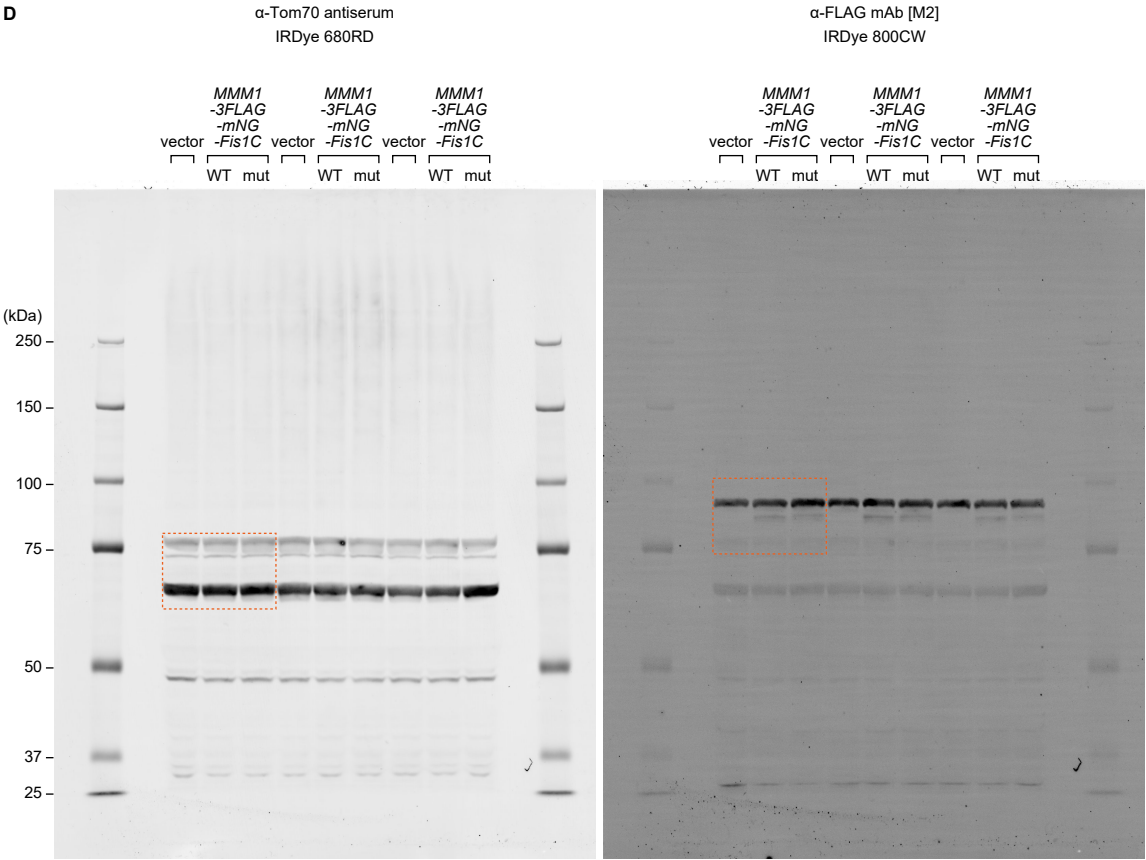

Supplement: SourceData F5 — is the source file for Fig. 5. [file jcb_202411196_sourcedataf5.pdf]

Source Data Supplementary Fig. 5

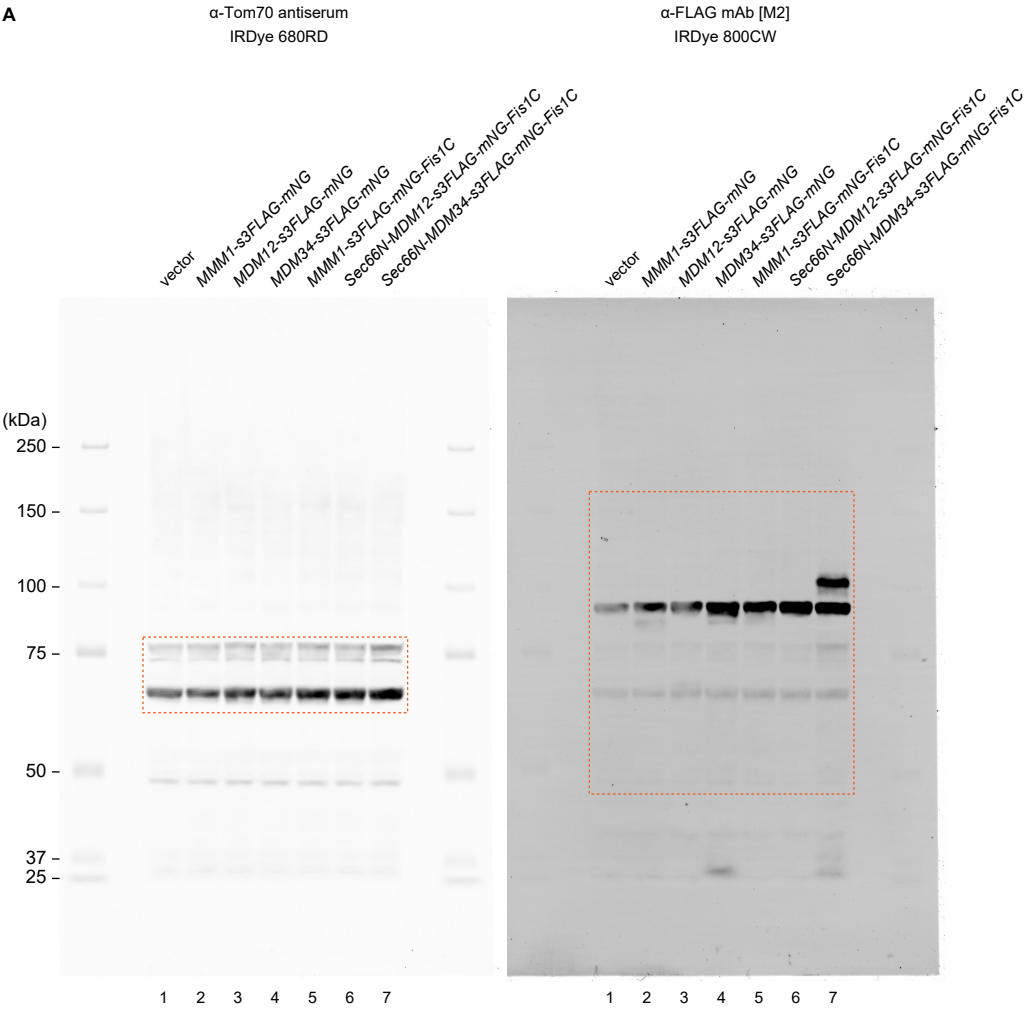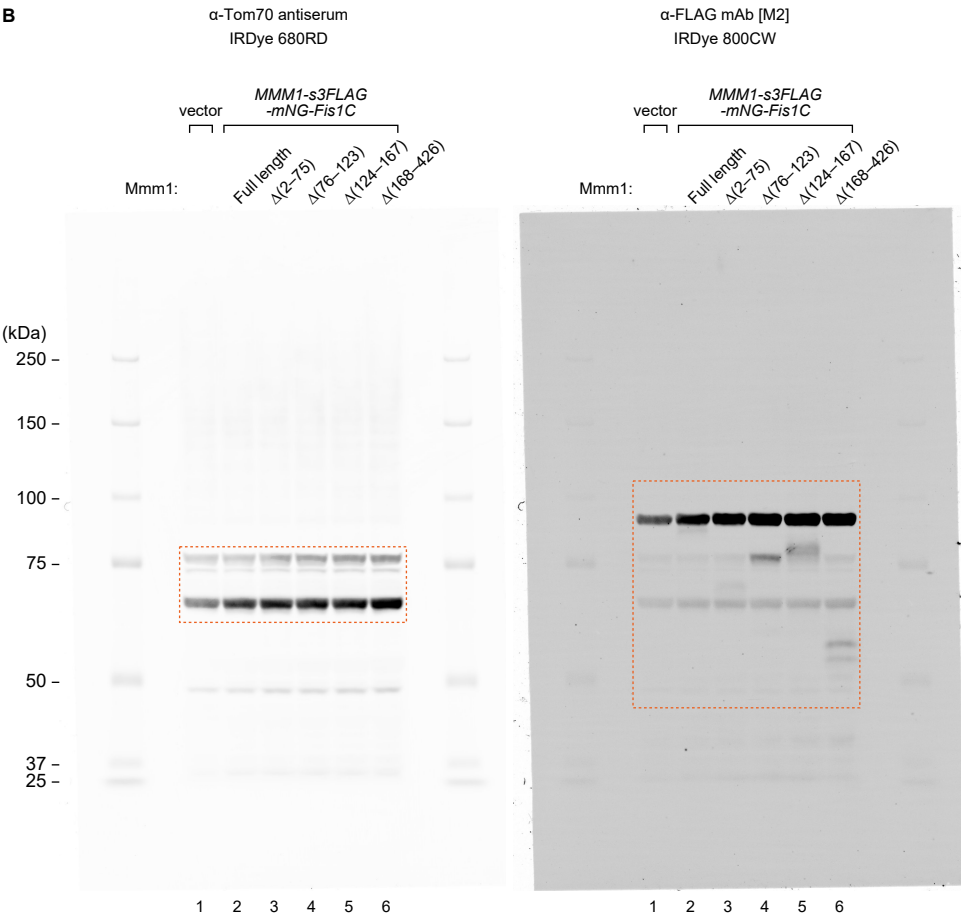

**C**

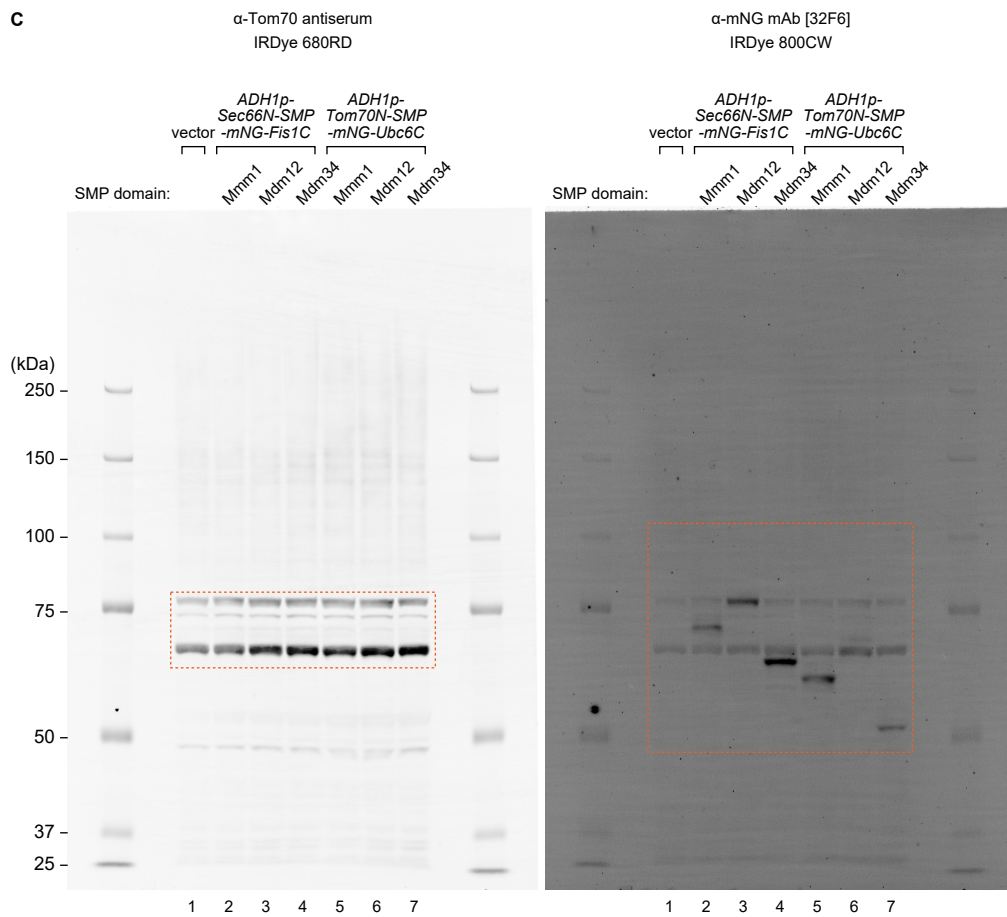

Supplement: SourceData FS5 — is the source file for Fig. S5. [file jcb_202411196_sourcedatafs5.pdf]
